# Bowel perforation due to chronic idiopathic megacolon: Case report and literature review

**DOI:** 10.1016/j.ijscr.2022.106777

**Published:** 2022-01-17

**Authors:** Mauro Giambusso, Pietro Fransvea, Gilda Pepe, Gabriele Sganga

**Affiliations:** Emergency Surgery and Trauma - Fondazione Policlinico “A. Gemelli” IRCCS, Rome, Italy; Catholic University of Sacred Heart, Rome, Italy

**Keywords:** Perforation, Chronic, Idiopathic, Megacolon, Case report

## Abstract

**Introduction and importance:**

Chronic idiopathic megacolon is a rare condition characterized by an irreversible distension of the colon in the absence of organic disease. The pathogenesis of this condition is still unclear and the data in literatures are not consistent.

**Case presentation:**

We report a case of an 87-years-old woman affected by bowel perforation in chronic idiopathic megacolon. The patient underwent an emergency subtotal colectomy with terminal ileostomy. The postoperative was uneventful. At the histopathological examination, no organic cause of megacolon was found, so a diagnosis of idiopathic megacolon was done.

**Clinical discussion:**

Idiopathic megacolon is difficult to diagnose due to the lack of specific clinical manifestations and pathological features. If not carefully investigated, can lead to severe complications such as perforation of the dilated bowel and subsequent peritonitis and sepsis, metabolic and electrolyte abnormalities. The protocols for management of IMC remains controversial. To achieve a good long-term outcome, early intervention is recommended.

**Conclusion:**

Early diagnosis of idiopathic megacolon is needed to perform the best therapeutic strategy and prevent complications, but further studies are needed.

## Highlights

• Chronic idiopathic megacolon is a rare condition with unclear pathogenesis and nuanced clinical manifestations.
• A limited number of cases are described in the worldwide literature.
• Further case studies are needed to reach early diagnosis and perform the best therapeutic strategy.

## 1 Introduction and importance

Megacolon is a condition defined as Loss of intestinal peristalsis and subsequent dilation of the colon in the absence of a mechanical obstruction. While the definition of megacolon has varied in the literature, most use a cecum measurement of greater than 12 cm in diameter to define megacolon [1]. Megacolon may be divided into two categories by acuity of onset [2], as follows: acute megacolon, including pseudo-obstruction, best known as Ogilvie's syndrome [3], [4], [5], probably secondary to an electrolyte/metabolic imbalance, and toxic megacolon [6], [7], [8], associated with systemic toxicity due to infectious colitis (*Clostridium difficile* pseudomembranous colitis, Salmonella enterocolitis) or inflammatory colitis (inflammatory bowel diseases); and chronic megacolon, which includes congenital, acquired, and idiopathic causes, due to an underlying neuropathic (Hirschsprung's disease [9], [10], chronic Chagas disease [11], [12]) or myopathic disorder (Duchenne's muscular dystrophy). Idiopathic megacolon is a rare condition in which the colon dilatation has no identifiable organic cause. These patients suffer from recurrent episodes of constipation, abdominal pain, distension and bloating starting in childhood or adolescence. Due to increased intraluminal pressures, these patients are at an increased risk of ulceration and then of perforation of the bowel wall. The pathogenesis of idiopathic megacolon is still unclear and the literature regarding the management of these patients is poor, therefore idiopathic megacolon represent a clinical challenge. This case report has been reported in line with the SCARE 2020 Criteria [13].

## 2 Case presentation

**Fig. 1:**
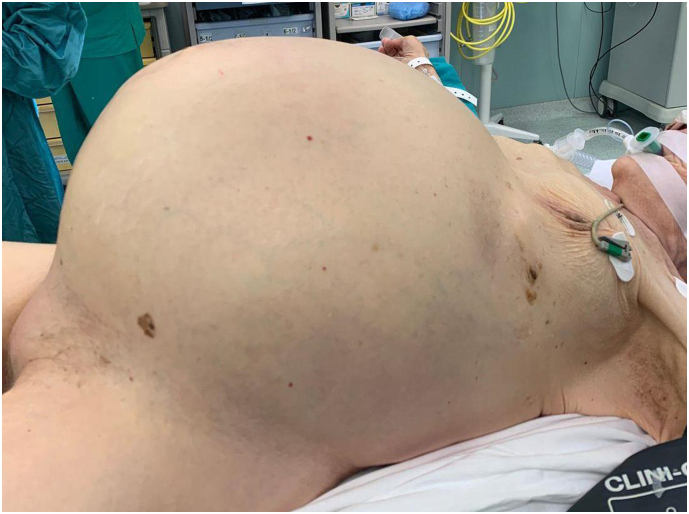
Physical examination showing massive abdominal distension.

**Fig. 2:**
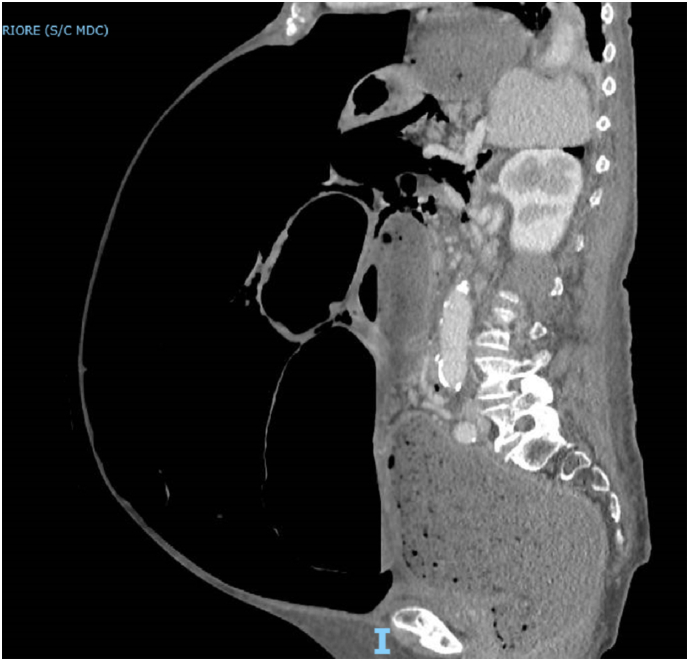
Sagittal TC-scan displaying free air in the abdomen with an important diffuse gaseous distension of the entire colon.

**Fig. 3:**
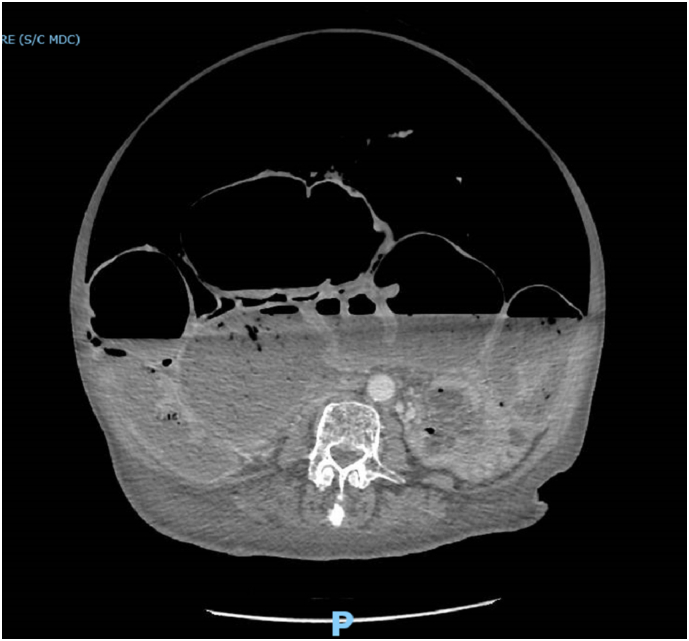
Axial TC-scan displaying free air in the abdomen with an important diffuse gaseous distension of the entire colon.

**Fig. 4:**
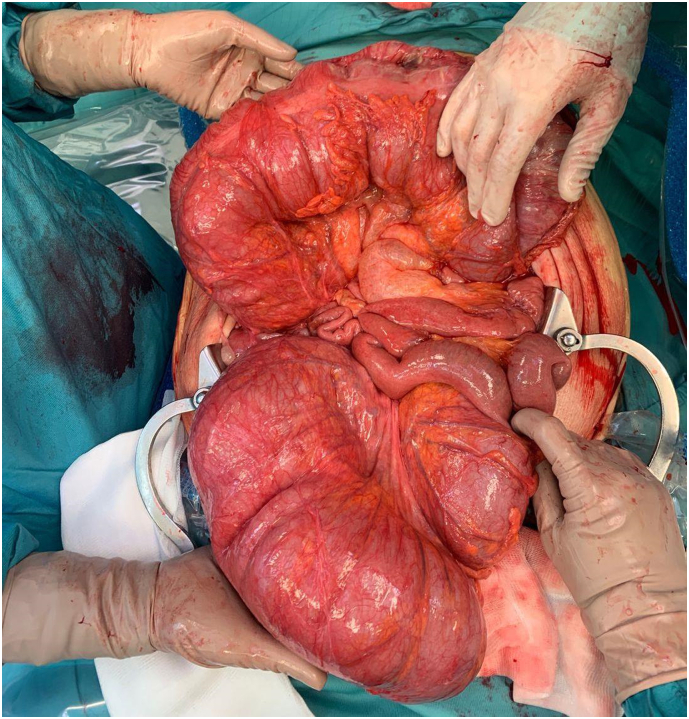
Intra-operative findings showing massive dilatation of the entire colon with no evidence of adhesions, volvulus, intussusception or torsion.

An 87-years-old female obese patient with Body Mass Index (BMI) of 31,25 kg/m^2^ (80 kg, 1,60 m) was admitted to our emergency department, in poor general conditions complaining of abdominal pain, bowel occlusion and a 1-week history of diarrhoea. She had a long history of chronic constipation. The patient suffered from arterial hypertension, hypercholesterolaemia and gallbladder microlithiasis. She denied any allergy. Past history was positive for hysterectomy due to uterine fibroma. Physical examination showed abdominal distension (Fig. 1) and a positive Blumberg sign. Laboratory findings on admission showed 14.7 g/dL haemoglobin, 17.0 × 109/L white blood cells, 1.18 International Normalized Ratio (INR), 40.4 s Activated Partial Thromboplastin Time (aPTT), 2.6 mmol/L serum potassium, 142 mmol/L serum sodium; the other laboratory values were unremarkable. Plain radiograph and computed tomography of the abdomen showed wide free air in peritoneum, associated to a moderate free air in retroperitoneum, predominantly localized in the peripancreatic region and that goes up through the esophageal hiatus in the posterior mediastinal region; the scan showed also an important diffuse gaseous distension of the entire colon, with air-fluid levels, a small intestine almost completely sagged, however no direct signs of bowel perforation were found (Figs. 2, 3). Due to the clinical condition the patient underwent an emergency laparotomy. On surgical exploration, the digestive tract displayed normal anatomy, with no evidence of adhesions, volvulus, intussusception, or torsion but did exhibit massive dilatation of all the colon from the recto-sigmoid junction to the ileocecal valve and perforation of the posterior wall of the transvers colon (Fig. 4). Due to these findings a subtotal colectomy with terminal ileostomy was performed. The postoperative course was uneventful and the patient was discharged in postoperative day 8th. The histopathological examination documented a 3 cm transverse colic continuous solution surrounded by fibro-granulocyte material and faecal residues; the submucosa showed marked hyperaemia and zonal sclerosis, the mucosa was characterized by diffuse light-moderate chronic inflammation; ganglion cells were well represented both in the myenteric and in the submucosal nervous plexus. At 1, 6 and 12 months follow-up the patient result in good condition.

## 3 Clinical discussion

Idiopathic megacolon is a rare type of megacolon that can lead to bowel perforation if left untreated. Our literature review found a small amount of data on idiopathic megacolon, while literature on congenital forms (Hirschsprung disease) is flourishing. The pathogenesis of idiopathic megacolon is still unclear. Besides abnormalities of the enteric nervous system, alterations in the function of intestinal smooth muscle cells and connective tissue elements might play an important role. Meier-Ruge et al. [14] analyzed 63 idiopathic megacolon resections between 1997 and 2004, resulting histologically characterized by a total atrophy of the collagenous tendinous connective tissue membrane of the myenteric plexus and the tendinous collagen fibre net of the muscolaris propria, involving collagen type III, missing in the muscolaris propria; the myenteric plexus was normal in the majority of patients, with no alteration about interstitial cells of Cajal, collagen type II and IV, smooth muscle actin, desmin and fibronectin, so the abolished peristalsis and distension of the colon of these patients was due to the atrophy of the tendinous fibre net. In addition, Autschbach et al. [15], according to the data of Meier-Ruge and colleagues, emphasize involvement of TFN (tendinous fibre network) atrophy of the muscolaris propria in the pathogenesis of idiopathic megacolon, however they underline how these results are basically descriptive and do not allow further conclusions concerning the involved cell types, molecular changes and possible genetic influences. Gattuso et al. [16] examined resected tissue from 24 patients who underwent surgery for idiopathic megarectum and from 6 patients who underwent surgery for idiopathic megacolon, observing a significant thickening of the enteric smooth muscle layers, while the architecture of the enteric innervation seemed to be intact either in the myenteric and submucosal plexuses. The natural history of this condition and optimal algorithm of diagnosis are far to be standardized. In our patient the clinical history was positive from chronic constipation with any infectious, metabolic, mechanical, endocrine-related etiology, these help us to formulate the diagnosis of idiopathic megacolon.

Early detection of signs of chronic idiopathic megacolon is fundamental to prevent complications and to perform a more conservative therapeutic strategy. In their systematic review, Cuda et al. [17] propose as diagnostic criteria for idiopathic megacolon the exclusion of organic disease by rectal biopsy or an intact anorectal inhibitory reflex, a sigmoid diameter approximately of 10 cm on abdominal X-ray or barium enema, symptoms including constipation, distension, abdominal pain and gas distress; other criteria, as the histology, revealed conflicting results: different enteric architectural and neurochemical abnormalities were found, as decreased concentration of interstitial cells of Cajal, diminished ganglia, diminished enteric neural densities or enteric smooth muscle hypertrophy possibly in association with normal enteric neuron histology. Wang et al. [18] review the current diagnostic criteria of chronic idiopathic megacolon, introducing the possibility to diagnose this condition when the radiological imaging is not conclusive through the intraluminal measurements of colonic compliance by measuring volume at 20, 32 and 44 mmHg distension; they investigate also different genetic associations with chronic acquired megacolon beyond childhood: in particular, the association of SEMA3F gene in a family with megacolon seemed to play a central role in the etiopathogenesis of this pathology. In their review, O'Dwyer et al. [19] analyzed clinical and motility features of 24 patients diagnosed with chronic megacolon, including colonic compliance and tone, adding on other important diagnostic criteria to this pathology. The cause of megacolon was idiopathic in 16 of them. High colonic compliance and low colonic tone were demonstrated, underlining the dysmotility that characterizes this condition.

Few cases of idiopathic megacolon are described in the literature. Generally, this disease affects more frequently adult population. The clinical presentation ranges from mild symptoms such as constipation, abdominal distension, to severe symptoms due to occlusive phenomena or intestinal perforation. Fransvea et al. [20] reported a case of a 43-years old obese man with Body Mass Index (BMI) 55.5 admitting to the emergency department for abdominal nausea starting 15 days earlier and vomiting; the abdomen was distended and painful, the Blumberg sign was positive and at Rx abdomen a distended colon was present. At laparotomy a giant megacolon was found, and a Hartmann procedure was performed; the anatomopathological examination documented no alteration of colon tissue. Differently, Liu et al. [21] described a rare case of an 11-years old boy that, after a 1-year history of intermittent constipation and abdominal distension occurred next to the ingestion of a large amount of fried sticky rice in 1 consumption, developed a clinical picture of megacolon, with a dilated colon and distal ileum radiologically measured up to 13 cm in maximum diameter. The patient was initially treated with gastrointestinal decompression and then saline enema, inefficiently, so he underwent surgical exploration, performing enteral decompression and a loop ileostomy. The examination of intraoperative frozen section of the rectum, colon and terminal ileum revealed mild neuron loss parallel decrease of nerve fibre density in the muscular layer and submucosal plexus in association of a mild chronic inflammatory cell infiltration in lamina propria. The positivity for S-100 protein, SYN, BCL-2, CD56, NSE and CGA led to the diagnosis of idiopathic megacolon. Intestinal perforation due to a delayed diagnosis is one of most common cause of mortality for patient affected by idiopathic megacolon, but not the only one. Hlavaty et al. [22] reported a case of two young patients (9 and 16 years-old respectively) died for idiopathic megacolon without perforation of the bowel wall. In both cases the patients suffered from a long history of chronic constipation and developed megacolon, dying the first after administration of a laxative, the second after a collapse. No identifiable cause of megacolon was formulated, so a diagnosis of idiopathic megacolon was elicited. The management of this clinical condition is still not unique ranging from conservative therapy with hydration, prokinetic drugs to invasive surgical procedure such Hartmann's resection. In the case report described by Anyaegbuna et al. [23], a 24-year-old man suffering from idiopathic megacolon was complicated with bowel perforation. The patient was first managed conservatively with oral laxative but due to a worsening of clinical condition CT-scan was performed showing a perforation of sigmoid colon, so an emergency laparotomy with a Hartmann procedure was performed. Anticipate worsening of the clinical evolution of this disease is fundamental in order to prevent fearful complications such as perforation, not always directly identified as in our case, that can quickly lead to death.

## 4 Conclusion

Chronic idiopathic megacolon appears to be clinically heterogenous, uncommon, and hence is often poorly managed. Further research and innovative molecular approaches are needed to get further insight into the etiology and pathogenesis of this disease in order to improve protocols for conservative therapy, and surgery for complicated cases.

## Funding

This research did not receive any specific grant from funding agencies in the public, commercial, or not-for-profit sectors.

## Ethical approval

The study is exempt from ethnical approval.

## Consent

Written informed consent was obtained from the patient for publication of this case report and accompanying images. A copy of the written consent is available for review by the Editor-in-Chief of this journal on request.

## Author contribution

Study concept and design: P. Fransvea.

Data Collection: M. Giambusso, G. Pepe.

Data Analysis and interpretation: M. Giambusso, P. Fransvea.

Writing the paper: M. Giambusso, P. Fransvea.

Revision: G. Sganga.

## Registration of research studies

Not needed.

## Guarantor

Pietro Fransvea MD is the Guarantor of the study.

## Provenance and peer review

Not commissioned, externally peer-reviewed.

## Declaration of competing interest

The authors declare no potential financial conflict of interest related to this manuscript.
